# Comparison of residual salivary fluoride retention using amine fluoride toothpastes in caries-free and caries-prone children

**DOI:** 10.1007/s40368-015-0220-x

**Published:** 2016-04-21

**Authors:** H. Nazzal, M. S. Duggal, M. B. Kowash, J. Kang, K. J. Toumba

**Affiliations:** Department of Paediatric Dentistry, Leeds Dental Institute, Leeds, UK; Hamdan Bin Mohamed College of Dental Medicine, Dubai, United Arab Emirates; Department of Oral Biology, Leeds Dental Institute, Leeds, UK

**Keywords:** Amine Fluoride, Children, Clearance, Fluoride, Saliva, Toothpaste

## Abstract

**Aim:**

This was to compare the salivary fluoride levels following tooth brushing with amine fluoride toothpastes containing three different concentrations of F (250 ppm F, 500 ppm F and 1250 ppm F) and to evaluate the effect of rinsing with water on the oral fluoride levels up to 90 min.

**Methods:**

A double blind randomised six-arm crossover study was conducted with 32 child participants. Patients were divided into two groups depending on their caries experience with caries-free group (*n* = 17, mean age = 72.9 months) and caries-prone group (*n* = 15, mean age = 69.6 months, mean dmfs = 12.3). Each participant brushed their teeth with a smear of dentifrice containing (250 ppm, 500 ppm and 1250 ppm F toothpastes) for 60 s. After spitting out the dentifrice/saliva slurry, participants either rinsed with water or did not rinse at all. Samples of whole mixed unstimulated saliva were collected at 0 (baseline), 1, 15, 30, 45, 60 and 90 mins post-brushing/rinsing.

**Results:**

After completing the study on residual fluoride concentration it was found that caries was not a significant variable (*p* = 0.567) while every other variable was (all *p* values <0.001). Time, toothpaste F concentration and rinse had significant effects (*p* < 0.001). In general, higher residual salivary F concentrations were found with increased F concentration in toothpastes and when no rinsing was performed after brushing.

**Conclusion:**

The results of this study support the current recommendation of using toothpastes with >1000 ppm F concentration in children with an increased caries risk in addition to spitting excess toothpaste with no rinsing following brushing.

## Introduction

Despite the dental profession’s continuous efforts in preventing dental caries, 31 % of 5 year olds and 46 % of 8 year olds were found to have obvious dental decay affecting their primary teeth according to the UK (2013) child dental health survey (Pitts et al. 2015). There are several prevention systems available, however, yet the use of fluoride (F) remains the most effective caries prevention tool, with overwhelming evidence of anti-cariogenic effect. Researchers have shown higher salivary fluoride levels in caries-free subjects in comparison to high caries subjects in both fluoridated and non-fluoridated areas (0.04 mg/L or greater in comparison to 0.02 mg/L or less) (Leverett et al. 1987; Shields et al. 1987; Toumba and Curzon 2001).

Several fluoride delivery systems are currently available; however, the use of fluoridated toothpastes is still the most widely used method. The use of fluoridated toothpastes was shown to elevate salivary fluoride levels and reduce dental decay (Walsh et al. 2010). A recent Cochrane review concluded that higher relative caries preventive effects were associated with increased toothpaste fluoride concentrations (Walsh et al. 2010). This effect, however, was significant for toothpastes containing 1000 ppm fluoride and above, with lack of evidence supporting toothpastes with lower fluoride concentrations. This led to the recommendation of using toothpastes with higher fluoride concentrations such as 1000 ppm fluoride in under 3 year olds and >1350 ppm fluoride for older children and those with increased caries risk (Department of Health and British Association for the Study of Community Dentistry 2014).

Regardless of the fluoride delivery method used, maintaining a low level of salivary fluoride (0.06 mg/L) at the plaque/saliva/enamel interphase has been shown to reduce dental caries (ten Cate 1997; Featherstone 1999). Therefore, it is important to study salivary fluoride clearance following tooth brushing and identify any associated factors influencing salivary fluoride retention.

In adults, fluoride clearance has been investigated by several researchers showing a link between salivary fluoride levels and fluoride concentration of the toothpaste (Duckworth and Morgan 1991; Duckworth and Stewart 1994). Such studies, however, have rarely involved children. Paul et al. (1993) reported a similar trend when using low fluoride toothpaste in 50 children between 7 and 9 years of age.

Salivary fluoride clearance following tooth brushing can also be affected by other factors such as mouth rinsing with water following brushing. Research conducted on adults has shown that rinsing habits might affect oral retention of fluoride following brushing (Duckworth and Morgan 1991).

Issa and Toumba in (2004) compared different types of toothpastes (amine fluoride, sodium monofluorophosphate and sodium fluoride) with different concentrations (250–1450 ppm F) followed by either rinsing with water or not rinsing, in a randomised controlled trial. The highest salivary F retention was observed with amine F toothpastes containing 1400 ppm F throughout the study (up to 120 min).

The following study was designed to compare the salivary fluoride levels following tooth brushing in young children with amine fluoride toothpastes containing three different concentrations of F (250 ppm F, 500 ppm F and 1250 ppm F) and evaluate the effect of rinsing with water on the oral fluoride levels up to 90 min. An additional aim was to evaluate whether there were differences in the oral salivary F clearance between caries-free and caries-prone children using amine F toothpastes.

The null hypothesis was that residual salivary F concentrations were not affected by toothpaste F concentration, water rinsing and the child’s dental caries experience.

## Materials and methods

Ethical approval was obtained from the local research ethics committee of the Leeds Teaching Hospitals NHS Trust and consent/assent was obtained from the person with parental responsibility and each child prior to recruitment. Thirty-two children comprising 17 in the caries-free group (mean age = 72.9 months, and dmfs = 0) and 15 in the caries-prone group (mean age = 69.6 months, and mean + SD dmfs = 12.3 ± 6.1) participated in this double blind randomised six-arm crossover study.

Participants used F-free toothpaste for 1 day prior to each leg of the study. Every participant in each group (caries-free and caries-prone) brushed their teeth with a smear (0.2 g) of dentifrice (250 ppm, 500 ppm and 1250 ppm F toothpastes) for 60 s. A computer generated randomisation table for the study arm order for each child was generated and kept independently of the researchers by another member of staff. Upon recruitment of patients, the independent staff member was contacted to inform the senior author and researcher with the study arm order. This was a double "blinded" study (identical coded tubes), therefore, neither patients nor clinicians were able to identify which fluoride concentration was used at each arm of the study. After spitting out the dentifrice/saliva slurry, participants either:

1. Rinsed with 10 mL of Leeds tap water (0.l ppm F) for 10 s (rinsing group).
2. Did not rinse at all (non-rinsing group).

This was supervised by one of the team members who was not involved with the collection and measurements therefore the assessors were "blind" to the rinsing effect. Samples of whole mixed unstimulated saliva were collected at 0 (baseline), 1, 15, 30, 45, 60 and 90 min post-brushing/rinsing. Salivary F levels were analysed immediately using an Orion F ion-specific electrode.

### Statistical analysis

The outcome variable (residual salivary fluoride concentration) was fitted with a linear mixed effect model with predictors as rinsing, caries experience, and concentration of toothpaste. The predictor effects were considered to be statistically significant at the 5 % level. The model included a random intercept to account for the repeated observations (over time at 0, 1, 15, 30, 45, 60, and 90 min) for each individual. All models were fitted using maximum likelihood estimation and backward elimination was used for model selection to obtain the parsimonious model. Statistical analysis was performed using IBM SPSS Statistics 21 software. A statistician, one of the authors of this manuscript (JK), performed the statistical analyses.

## Results

After obtaining the full model, it was found that dental caries was not a significant variable (*p* = 0.567) while every other variable was (all *p* values <0.001). Therefore, dental caries was excluded in the new model. Time, toothpaste F concentration, and water rinsing had significant effects (*p* < 0.001). Interaction effects between any two of these variables were also significant. The three-way interaction between time, toothpaste F concentration and rinse was also significant.

**Fig. 1:**
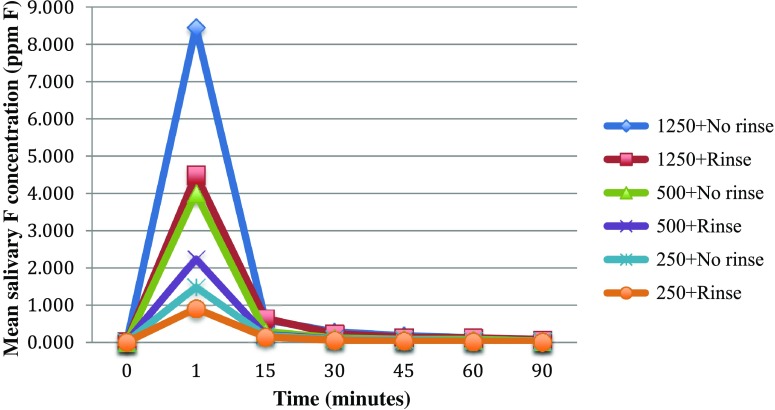
Line graph showing mean residual salivary fluoride concentrations after brushing with three amine fluoride toothpastes (250, 500 and 1250 ppm F) with rinsing and no rinsing over time

**Fig. 2:**
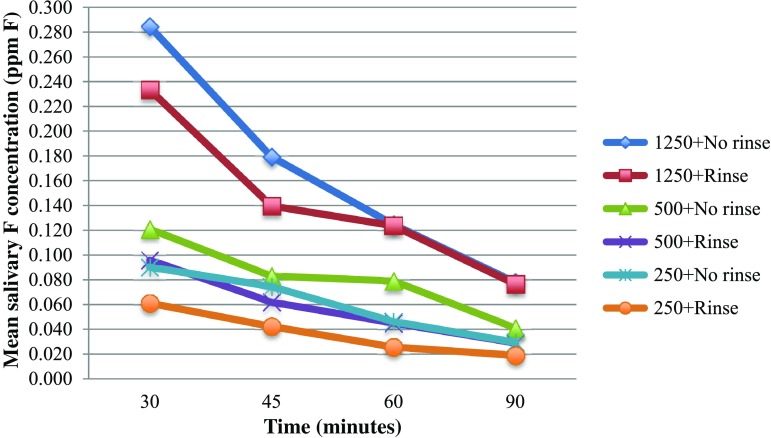
Line graph showing mean residual salivary fluoride concentrations after brushing with three amine fluoride toothpastes (250, 500 and 1250 ppm F) with rinsing and no rinsing over time in relation to the minimum anti-cariogenic salivary fluoride concentration of 0.06 ppm F

Figures 1 and 2 show a similar pattern of salivary F clearance with time regardless of toothpaste F concentration. In general higher residual salivary F concentrations were found with higher F concentration in the toothpaste and when no rinsing was performed after brushing. Residual salivary F concentrations remained over the 0.06 ppm salivary F threshold for caries prevention up to 90 min with 1250 ppm F toothpaste regardless of rinsing. The same pattern was observed for toothpastes containing 500 ppm F with no rinsing. The 250 ppm F containing toothpaste with rinsing dropped under the threshold after only 30 min.

**Fig. 3:**
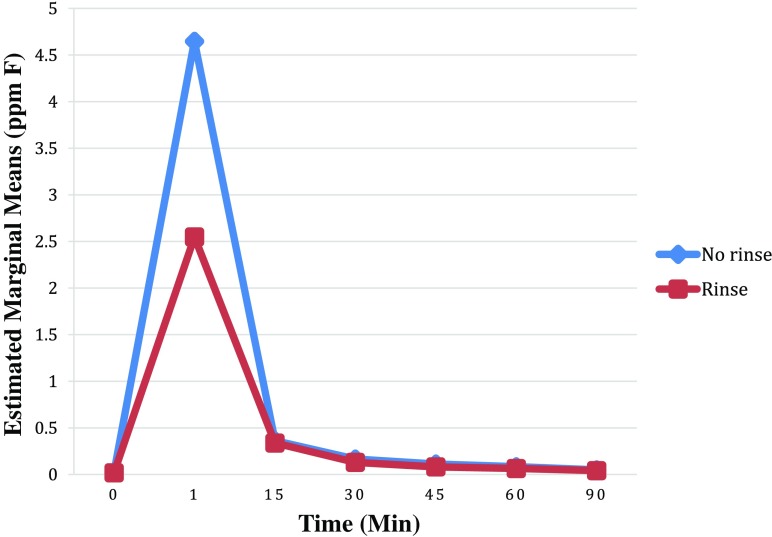
Graph showing estimated marginal means of salivary fluoride concentrations between rinsing and no rinsing (excluding toothpaste fluoride concentration and caries as variables) at 0–90 mins

Figures 1 and 3 show that for any given toothpaste, the residual salivary fluoride levels were lower after rinsing with water.

The effect of toothpaste F concentration showed the lowest *p* value (<0.001) and therefore should be considered the factor with the most significant effect in the model. There are significant differences when comparing toothpastes with different F concentrations against each other (1250–250, 1250–500, 250–500 ppm F, all *p* values <0.001). A higher residual salivary fluoride concentration was measured when using toothpaste with higher F concentration.

## Discussion

### Toothpaste F concentration

The use of fluoridated toothpastes has been associated with reduction in dental caries (Walsh et al. 2010) with higher caries prevention associated with higher F concentration in toothpastes. The use of high F containing toothpastes, however, is associated with a higher risk of fluorosis (Atia and May 2013), therefore, assessing the anti-cariogenic effect and salivary F retention of toothpastes with lower F concentrations is indicated.

The use of 1400 ppm amine F containing toothpastes was shown to increase salivary F levels up to 120 min in children (Issa and Toumba 2004). Similar results were found in adults following use of amine fluoride toothpaste (1250 ppm F for 90 min) (Attin and Hellwig 1996). Therefore, this study compared three different amine F concentrations with lower F concentrations (250, 500 and 1250 ppm F). The pattern of salivary F concentrations over time was similar for all toothpastes used which is consistent with the work by Issa and Toumba (2004). Residual salivary F concentrations were highest after 1 min regardless of toothpaste used followed by steep reduction up to 15 min. This was then followed by gradual reduction up to 90 min regardless of toothpaste F concentration.

Toothpaste F concentration was the variable showing the most significant effect in the model, used to analyse the results of this study. Furthermore, salivary F concentrations remained over the 0.06 ppm salivary F threshold for caries prevention up to 90 min with 1250 ppm F toothpaste regardless of rinsing experience. Therefore, based on the results of this study using 1250 ppm F containing toothpaste is recommended.

Based on the pattern of F clearance, seen in all types of toothpaste used in this study, salivary F concentrations gradually decreased to a level under the recommended 0.06 ppm F. Therefore regular brushing is recommended in order to maintain salivary F concentrations over the recommended levels.

Although no high quality evidence is available on the effect of brushing before or after meals, in vitro evidence showed less mineral loss with brushing before meals (Toumba 2012). The maximum protection of F toothpastes in this study seems to be within the first 1–15 min and therefore for F to exhibit the maximum anti-cariogenic effect, tooth brushing just before a cariogenic challenge is recommended. In addition, Sjögren and Birkhed (1994) showed 12–15 times reduction in residual F levels immediately following eating and when brushing was performed before eating which could indicate the utilisation of F in cariogenic areas.

### Rinsing after tooth brushing

This study showed that, in general, rinsing the mouth with water after brushing was associated with lower residual salivary F concentrations which was consistent with the studies conducted on adults (Attin and Hellwig 1996). The use of 500 ppm F containing toothpaste followed by no rinsing was associated with residual salivary F levels higher than 0.06 ppm F up to 90 min, which was similar to the 1250 ppm F toothpaste. The use of 1250 ppm F containing toothpaste, however, might provide longer protection than 90 min (Fig. 2). Figure 2 shows no effect of rinsing on mean salivary F concentrations after 60 min, however, in the statistical model used herein showed rinsing to have a significant effect regardless of F toothpaste concentration.

## Conclusion

The null hypothesis is therefore rejected with regards to the effect of higher F containing toothpaste and rinsing following tooth brushing on residual salivary F concentrations. The null hypothesis is accepted with regards to the effect of caries experience on residual salivary F concentrations.

The results of this study, therefore, support the current recommendation of spitting excess toothpaste with no rinsing following brushing (Department of Health and British Association for the study of Community Dentistry 2014). In addition, this study supports the recommendation to use toothpastes with >1000 ppm F concentration in children with an increased caries risk.
